# Odintifier - A computational method for identifying insertions of organellar origin from modern and ancient high-throughput sequencing data based on haplotype phasing

**DOI:** 10.1186/s12859-015-0682-1

**Published:** 2015-07-28

**Authors:** Jose Alfredo Samaniego Castruita, Marie Lisandra Zepeda Mendoza, Ross Barnett, Nathan Wales, M Thomas P. Gilbert

**Affiliations:** Centre for GeoGenetics, The Natural History Museum of Denmark, University of Copenhagen, Øster Voldgade 5-7, Copenhagen, DK-1350 Denmark

**Keywords:** Ancient DNA, High-throughput sequencing, Mitochondrial assembly, Numt, Nupt, Odin, Phasing

## Abstract

**Background:**

Cellular organelles with genomes of their own (e.g. plastids and mitochondria) can pass genetic sequences to other organellar genomes within the cell in many species across the eukaryote phylogeny. The extent of the occurrence of these organellar-derived inserted sequences (odins) is still unknown, but if not accounted for in genomic and phylogenetic studies, they can be a source of error. However, if correctly identified, these inserted sequences can be used for evolutionary and comparative genomic studies. Although such insertions can be detected using various laboratory and bioinformatic strategies, there is currently no straightforward way to apply them as a standard organellar genome assembly on next-generation sequencing data. Furthermore, most current methods for identification of such insertions are unsuitable for use on non-model organisms or ancient DNA datasets.

**Results:**

We present a bioinformatic method that uses phasing algorithms to reconstruct both source and inserted organelle sequences. The method was tested in different shotgun and organellar-enriched DNA high-throughput sequencing (HTS) datasets from ancient and modern samples. Specifically, we used datasets from lions (*Panthera leo ssp.* and *Panthera leo leo)* to characterize insertions from mitochondrial origin, and from common grapevine (*Vitis vinifera)* and bugle (*Ajuga reptans*) to characterize insertions derived from plastid genomes. Comparison of the results against other available organelle genome assembly methods demonstrated that our new method provides an improvement in the sequence assembly.

**Conclusion:**

Using datasets from a wide range of species and different levels of complexity we showed that our novel bioinformatic method based on phasing algorithms can be used to achieve the next two goals: i) reference-guided assembly of chloroplast/mitochondrial genomes from HTS data and ii) identification and simultaneous assembly of odins. This method represents the first application of haplotype phasing for automatic detection of odins and reference-based organellar genome assembly.

**Electronic supplementary material:**

The online version of this article (doi:10.1186/s12859-015-0682-1) contains supplementary material, which is available to authorized users.

## Background

It is now widely appreciated that DNA sequences from a cellular organellar genome can be inserted and integrated into another organellar genome in many different species [1]. For example, in plants, sequences of the chloroplast genome can be inserted into both the mitochondria (mtpt) [2, 3] and nucleus (nupt) [4, 5], while mitochondrial DNA (mtDNA) can be inserted into the nucleus (numt) [5]. Many numt sequences have been reported in fungi, insects, and vertebrates [6] (including fish [7], birds [8], amphibians [9], and reptiles [10, 11]). These sequences can even be copied to other loci multiple times in the new hosting genome [12]. The mechanisms underlying this DNA transfer process are not yet completely understood [8, 13], but studies suggest that such integrations are enabled by different genetic and environmental factors during the repair of double strand breaks by non-homologous end joining [14–16]. The extent of the DNA insertions can be very large, both regarding the number of occurrences as well as the length of the insertion. For example, those in the human nuclear genome represent the entire mitochondrial genome [17].

Although the existence of *o*rganellar-*d*erived *in*serted sequences (hereby referred to as “odins”) was first accepted more than 30 years ago [1], the techniques that are currently available for the identification of such sequences are not suited to the complexity and size of high-throughput sequencing (HTS) datasets. However, accurate identification of odins is extremely important because if not accounted for, they can confound the results of sequence-based analyses that rely on the principle of orthology [18]. For example, because the rate of evolution of nuclear inserted sequences is different to those of the homologous source organellar sequences, inaccurate phylogenetic reconstructions [19] or spurious cryptic species (morphologically identical but genetically different species) can be inferred [20]. Furthermore, population genetic studies such as demographic profiles can be benefited from the identification of odins [21].

Traditionally, laboratory methods to identify odins include mitochondrial enrichment [22–24], PCR on diluted extracts [25], PCR amplification from RNA derived from coding DNA [26, 27], and nested PCR [22, 23]. Since mtDNA and plastid DNA (ptDNA) is usually found at higher copy number than nuclear DNA, the integration of careful steps to identify odins into automatic computational methods for organellar sequence assembly has not been deeply explored. However, the supposition that the true organellar sequence will ultimately be determined is challenged by cases of preferential amplification [28] or primer binding bias during the PCR step [29]. Given this, current computational methods for dealing with odins may miscall single nucleotide variants (SNVs) or indels, leading to a possible erroneous consensus organellar sequence. Traditional computational methods for identification of odin-derived SNVs and thus assembly of accurate organellar sequence rely on identifying changes in the phylogenetic position of the closest reference mitogenome [29], changes in the structure of rDNA [30] and misplaced stop codons [31, 32]. Following the advent of HTS, several methods have been designed that take advantage of the amount of mapping reads. Examples of these coverage-based methods are those that simply retain the nucleotide that is present in the highest copy number (majority count, MC) or quality in each position [24, 33], or stricter strategies that retain the nucleotide if it is present at a minimum ratio of 2/3 [33]. Another method is based on either masking known numts or disregarding putative numts by using only the unique mapping reads if both nuclear and organellar genomes are available [34]. Yet another method uses *de novo* assembly of the organellar genome, which is then manually curated [35].

Each of these methods has drawbacks. Laboratory methods are difficult, if not impossible, to apply to DNA where a reference genome is lacking, or where the DNA and/or cellular membranes are sufficiently degraded so as to preclude techniques such as nested PCR and organellar enrichment, such as in ancient DNA (aDNA) samples [36, 37], where numts have also been documented [38, 39]. Even in modern samples with well-preserved DNA, the consensus sequences obtained by MC may be inaccurate if there was library construction or amplification bias [40]. Available computational methods are limited to odins producing stop codons or changes in structure in coding or tRNA genes, thereby missing some portions of the genomes. Methods based on masking numt sequences or using only reads mapping uniquely to a genomic reference that contains the nuclear and the mitochondrial genomes together are naturally limited to analysis of data from well-studied organisms. Also, *de novo* sequence assembly is a rather unsupervised method of producing a consensus sequence that has a high risk of having chimeric regions containing both odin and source organellar sequences. Lastly, these computational methods do not allow for the simultaneous identification and assembly of odins, which is suboptimal given their possible use in evolutionary studies. For example, as relics of ancient mtDNA, these pseudogenes can be used for inferring ancestral states or rooting mitochondrial phylogenies [41]. Additionally, when numerous and selectively unconstrained, numts can be used for the study of spontaneous mutation in nuclear genomes [6, 42].

We present a computational method, odintifier, for the identification and reconstruction of odins based on haplotype phasing of HTS data [43]. Our method is the first application of haplotype phasing for automatic detection of odins and reference-based organellar genome assembly. As the method requires only an organellar genome of the species or a close relative, it can be applied to datasets from both ancient as well as modern non-model organisms. To aid in the time consuming manual curation that a *de novo* assembly would require, the method can also be used to assess the organellar genome obtained from a previous assembly and at the same time identify any present region source of odins.

**Fig. 1:**
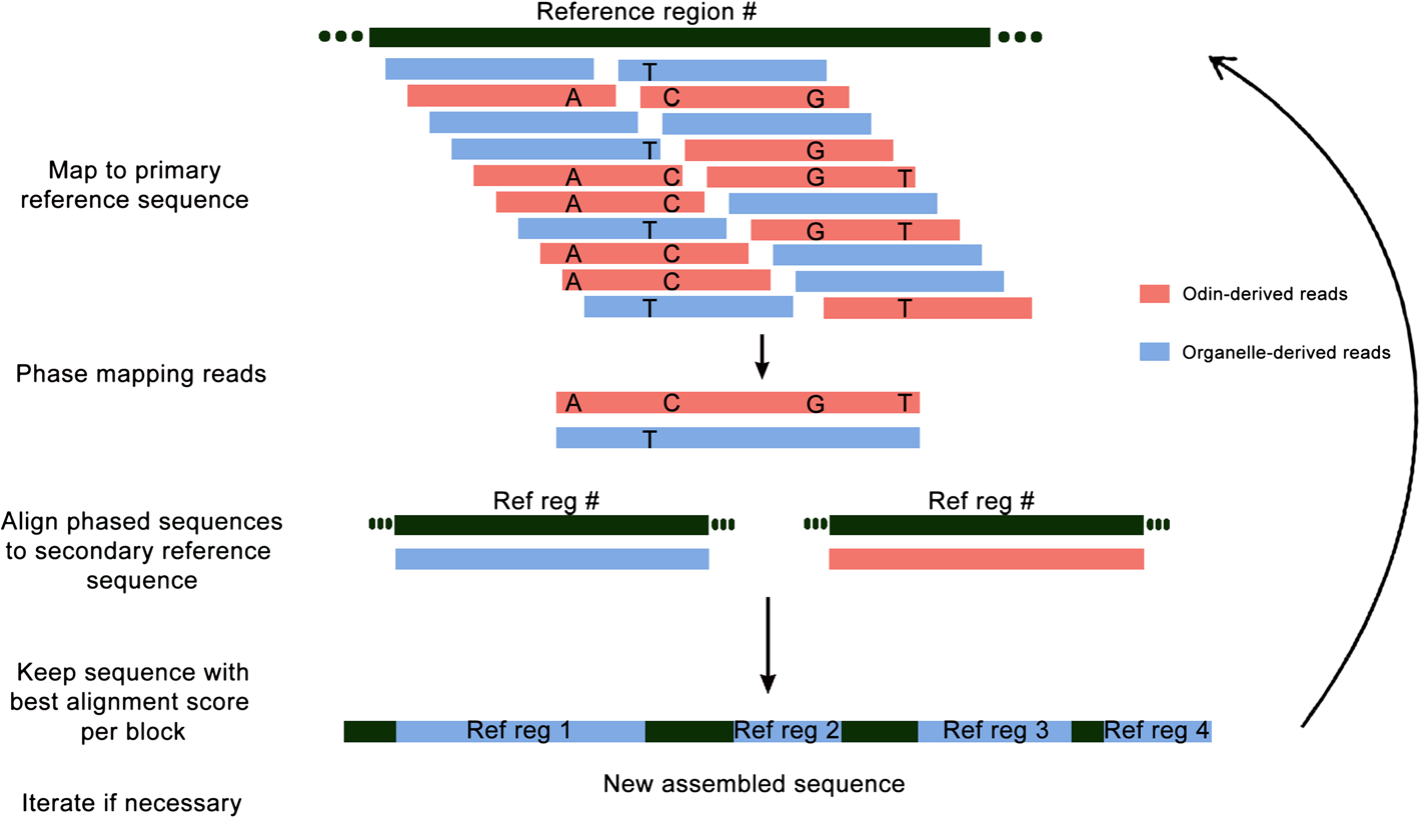
Workflow scheme. First the reads are mapped to a reference sequence, called primary reference. Some of the mapping reads can be used to identify haplotype regions and link the reads corresponding to the odin and the source organellar sequence. Afterwards, a sequence is assembled for both the odin and the source organellar sequence for each region. Each of the assembled sequences is compared to a new reference sequence, called secondary reference, which may or may not be the same as the primary sequence. For each region, the sequences are assigned to be odins or source organellar based on the alignment score to the secondary reference. Finally, an organellar genome sequence is assembled, where the regions previously identified to contain odins now contain the chosen sequence from the alignment. The steps can be repeated in an iterative fashion using the resulting assembled sequence as the new primary reference sequence

Broadly speaking, a haplotype is the sequence of nucleotides along a single chromosome, and haplotype phasing algorithms assign a genotype to a chromosome. To date, the application of haplotype phasing has largely been limited to studying the evolution of haplotypes [44–47] and genomic diversity between populations [48, 49], as well as for detecting associations among individuals [50–52] or to diseases [53–55]. While the organellar genome is haploid, the odin can be considered to be polyploid, with one copy being from the source organelle and one or more being from the host organelle. For example, a region from the mitochondria (the source organelle) would be one haplotype, and the sequence from that mitochondrial region inserted into the nucleus (the host organelle) would be the other haplotype. Thus, there will be haplotype informative reads [56] (i.e. reads that cover the heterozygous sites arisen by the odins) that can help separate the inserted and the source sequences (Fig. 1). Thus, the application of phasing in odintifier allows to achieve the next two main goals: i) reference-guided assembly of chloroplast/mitochondrial genomes from HTS data and ii) identification and simultaneous assembly of odins.

## Results

### Bugle analysis

**Fig. 2:**
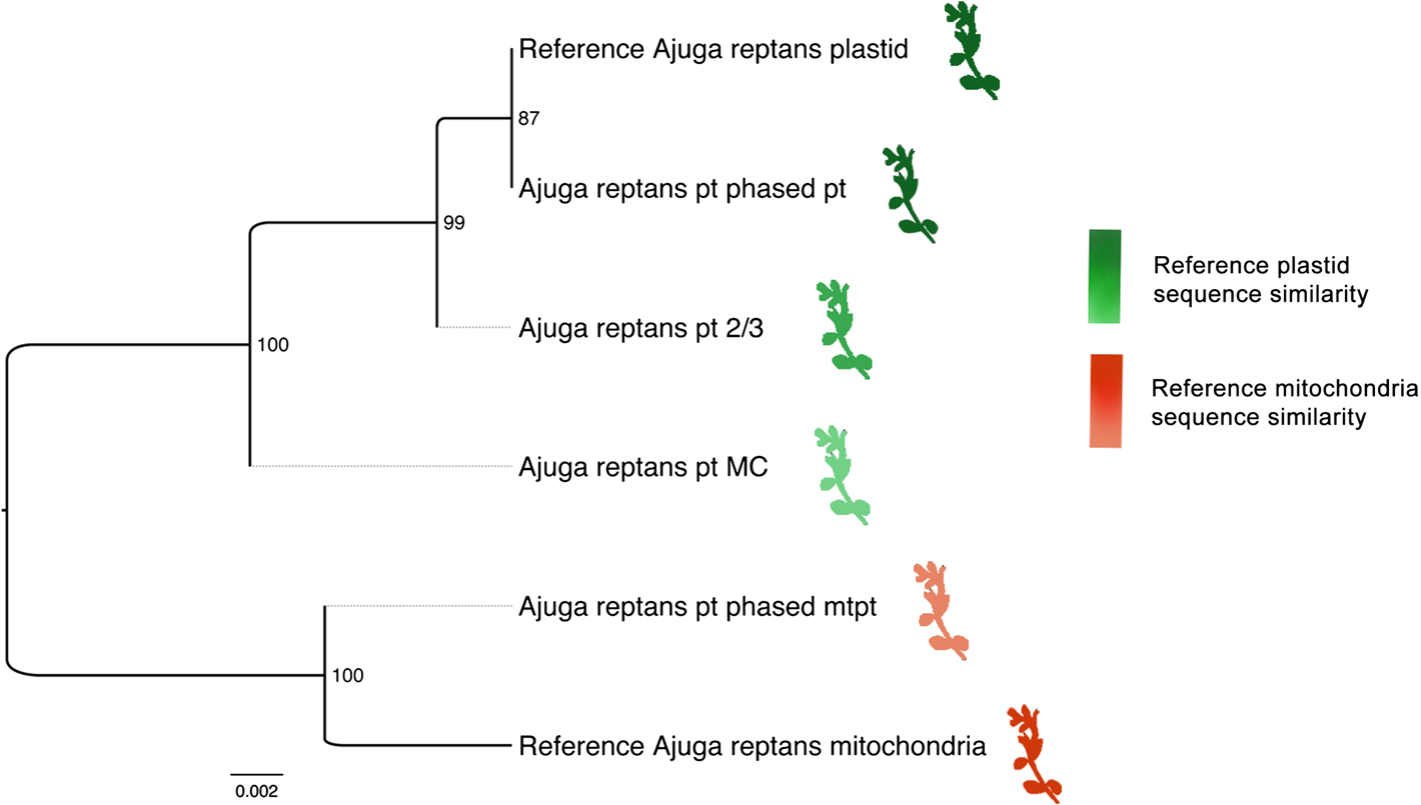
Bugle phased block. Sequence similarity dendrogram of the reconstructed sequences from the region on the plastid genome identified to contain odin-derived reads. The reconstructed plastid sequence (i.e. the organellar genome region source of the odin) is shown in a non-quantitative green gradient according to the similarity to the plastid reference (NC_023102.1), the reference mitochondrial region host of the odin is shown in red (NC_023103.1), and the reconstructed odin mtpt is shown in orange. The MC and 2/3 methods are not able to simultaneously reconstruct the odin sequence using only a chloroplast reference sequence, thus the missing MC and 2/3 mtpt branches in the dendrogram

In order to test the method on a plant sample with the goal of determining the quality of a *de novo* assembled plastid sequence, we used the dataset and the assembled chloroplast from Zhu et al. (2014) [35] and mapped the reads back to the assembled reference. Only one region of the mapping was identified as containing reads from a mitochondria-derived odin (Additional file 3: Figure S1). The chloroplast genome reference sequence reported by Zhu et al. does not contain nucleotides from the pseudogene. The sequence similarity dendrogram of the assembled sequences with the three tested methods (MC, 2/3, and odintifier) positioned the sequence obtained by our phasing approach closest to the reference sequence, while the MC method produced the least accurate sequence (Fig. 2).

The pseudogene is located in the region 53630–55078 (as in NC_023102.1), which includes a fraction of the ribulose-bisphosphate carboxylase gene (*rbcL*). In order to test for missense or non-synonymous SNVs, which might lead to erroneous results if used for other kinds of analyses (e.g. phylogenetic or selection analyses), we translated the sequences reconstructed with the three different methods. The gene sequence obtained using the MC method contains six miscalled bases, leading to five changed amino acids, while that generated following the 2/3 rule contains two miscalled bases, leading to one wrong amino acid when compared to the reported *rbcL* protein sequence (YP 008964042.1 [35]). Taken together, this dataset showcased that odintifier can be successfully applied to assess an organellar genome previously obtained from a *de novo* assembly and at the same time identify any present region with SNVs that can be due to odins or heteroplasmy.

### African lion analysis

**Fig. 3:**
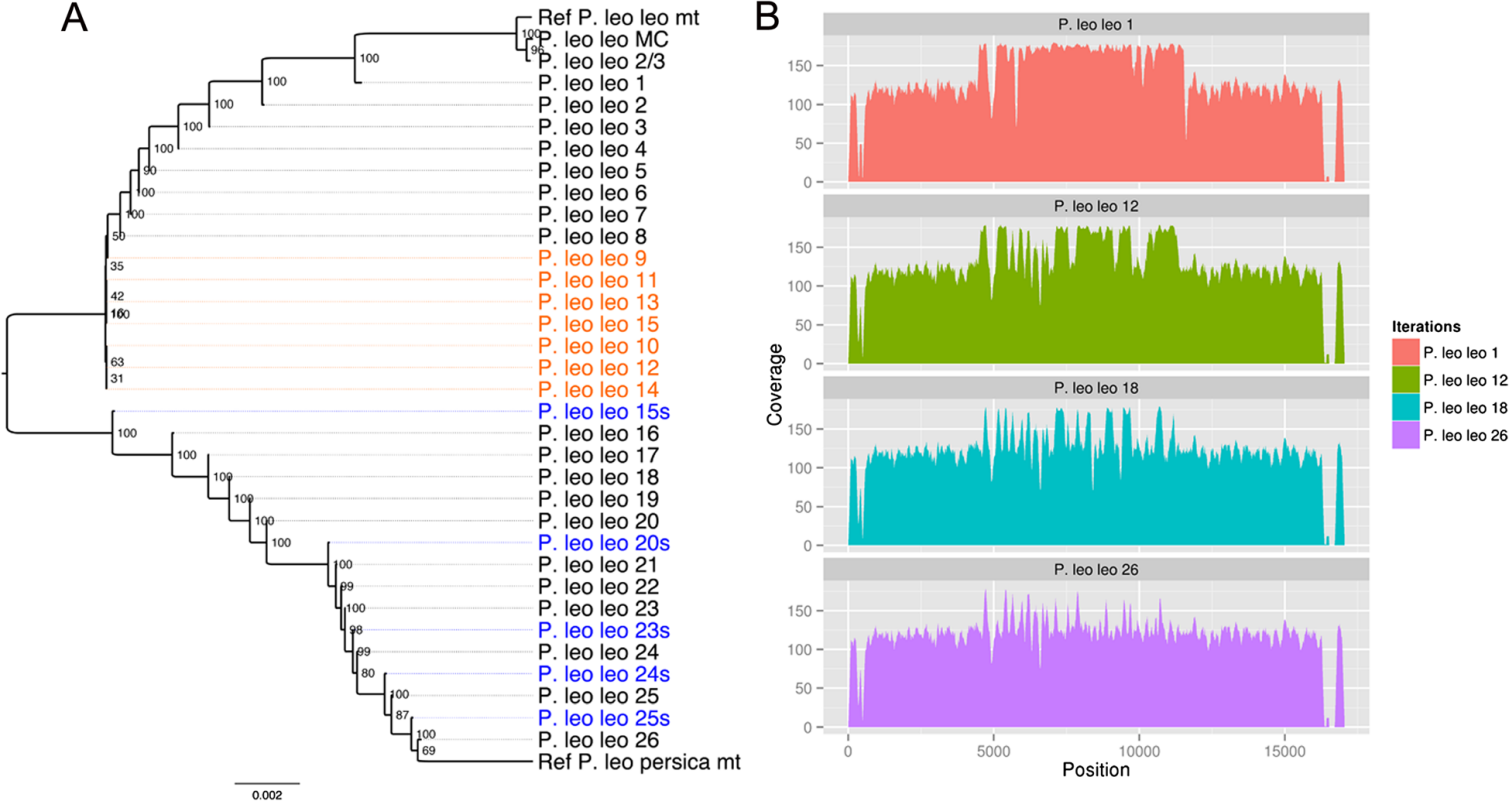
African lion mitochondrial sequence assembled through iterations. **a** Sequence similarity dendrogram of the sequences reconstructed on the different iterations of odintifier using as reference the mitogenome KF776494.1 and NC_018053.1 excluding the control region. Black labeled iterations represent the use of odintifier with GenomeAnalysisTK for the phasing step. Orange labeled iterations are those in which the resulting sequence from using GenomeAnalysisTK for the phasing converged to almost the same unresolved sequence. Blue iterations implement the use of samtools for the phasing step to separate the reads by allele, greatly enhancing the resolution of multiallelic blocks and overcoming the convergence state. **b** Coverage on the reconstructed African lion sequences obtained from iterations 1, 12, 18, and 26

In order to test the method on an assembled mitochondrial sequence from a feline species, a taxonomic group that has been observed to carry large numbers of numts [57, 58], we used the African lion assembled mitochondria and dataset from Ma and Wang (2014) [59]. We applied our method and performed a total of 31 iterations of mapping and phasing with odintifier until the sequences converged, i.e. did not differ from one iteration to the next. Based on a sequence similarity dendrogram of the pair-wise distance analysis of the entire mitochondria excluding the control region, we observed that the recovered sequences are significantly different. Only the mitogenomes reconstructed with coverage-based methods cluster to the original African lion assembly that contains odin-derived SNVs, while the sequences from the different iterations of the phasing method are more similar to the mitochondrial reference of *P. leo persica* on each progressing iteration (NC_018053.1 [60]) (Fig. 3a).

Interestingly, while using odintifier with GenomeAnalysisTK for the phasing step we noted that the reconstructed mitogenome sequence appeared to converge after the 9th iteration (i.e. it did not change significantly between iterations 9 and 15). However, still many regions were not fully resolved. At this point, switching odintifier to the samtools-based approach (see Methods – Method development section) produced an immediate effect, as in subsequent iterations the resulting sequence continued to improve.

Notably, when comparing the depth of coverage across the mitogenome on the reconstructed sequences in the 31 iterations, we observed that the coverage became more homogeneous as more iterations were performed (Fig. 3b). This is particularly evident over the regions of multiple alleles that had a higher coverage in comparison to other regions without many alleles. By the final iteration, the coverage was largely uniform, except in the control region, which remained uneven throughout all 31 iterations.

The assembly of the mitochondria from a dataset with various odins proved that odintifier can be successfully applied following various iterations in order to fulfill its main first goal of assembling the organellar genome. However, the use of iterations with a diploid phasing algorithm on a dataset with various odin-derived haplotypes from the same region, i.e. containing more than two alleles, precludes accurate simultaneous assembly of the odin sequences. In spite of this, the positions of the odin source region can still be obtained in each iteration, thus partially achieving the second goal of simultaneous identification and assembly of the odin sequence (Additional file 3: Table S1).

### Ancient lion analysis

**Fig. 4:**
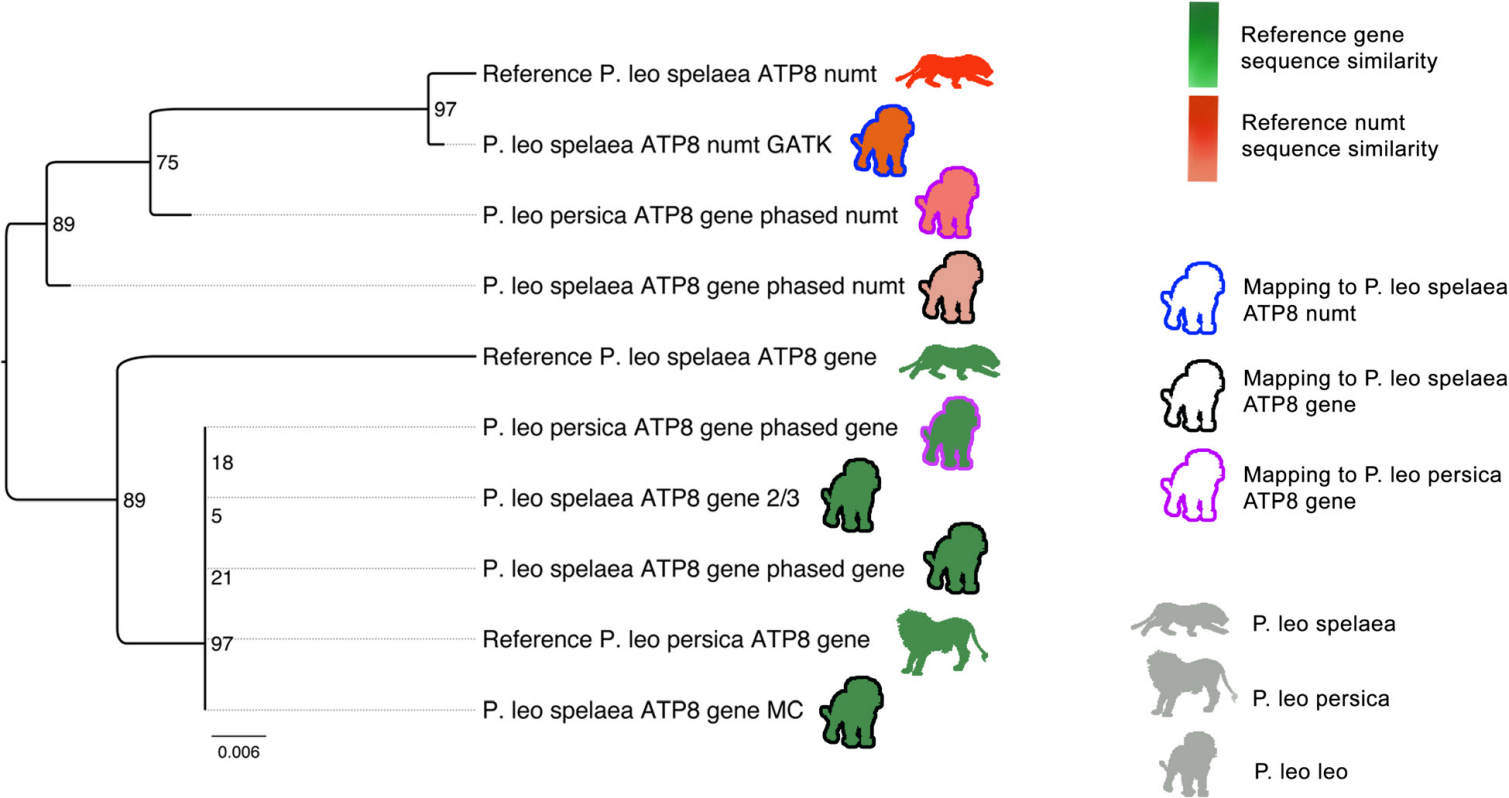
Ancient lion *atp8* sequences. Sequence similarity dendrogram of the odin numt (*red silhouettes*) and mitochondrial (*green silhouettes*) reconstructed *atp8* sequences using coverage-based methods (blue contour) and phasing. Two available mitochondrial gene reference sequences (NC_018053.1 and DQ318556.1) and one reference numt sequence (DQ318555.1) were used

In order to explore the potential of odintifier on aDNA datasets, the lion aDNA dataset was initially mapped against two sequences of the well-characterized *atp8* gene (NC_018053.1 [60] and DQ318556.1 [61]) and the corresponding odin-derived *atp8* pseudogene sequence (DQ318555.1). After confirming that both the gene and the pseudogene sequences were present (Additional file 3: Figure S2), we applied odintifier and were able to reconstruct the gene and the pseudogene sequences in a single iteration (Fig. 4). In contrast to the previous dataset, the performance of all three tested methods was equally good, as all of them clustered together with the two reference sequences with very few differing positions between them.

Only odintifier is able to simultaneously reconstruct both the pseudogene and the gene sequence using the DQ318556.1 gene sequence as reference. Thus, in order to compare the performance of our phasing-based method to the coverage-based methods on the reconstruction of the numt, we obtained a coverage-based pseudogene sequence using the DQ318555.1 numt sequence as reference. Given that the SNVs filtered by the 2/3 and MC thresholds were the same, only one coverage-based sequence was reconstructed with GenomeAnalysisTK. All assembled numt sequences clustered together with the corresponding numt reference sequence. The assembly of the *atp8* gene and the simultaneous assembly of the odin-derived *atp8* pseudogene showed that odintifier can achieve its two goals even when used with datasets of very short read lengths, such as those of aDNA.

### Grapevine analysis

Grapevine has some of the highest known proportions of ptDNA inserted into the mitochondria [62], thus the dataset from the grapevine represents an extremely challenging test case for the assembly of the plastid genome and the odins. In order to determine the extent of the complexity of the dataset and to analyze the 1impact of the mapping stringency on the assembly methods, we applied the coverage-based methods and a single iteration of odintifier with four different mapping stringencies (relaxed, default, stringent, and very stringent) and manually characterized the 334 identified differing positions of the reconstructed sequences compared to the reference.

We found that the nucleotides incorrectly called by odintifier are mainly due to two reasons. (1) The lack of haplotype informative reads on the bam file resulting from strict mapping parameters and leading to single SNVs (Additional file 3: Figure S3). (2) Blocks containing multiple alleles, which cause erroneous boundaries of the phased blocks (chimeric blocks) (Additional file 3: Figure S4) and various SNVs that confound the diploid phasing algorithm so that the two phased sequences are not correct (Additional file 3: Figure S5). Different from biallelic blocks, multiallelic blocks were better assembled with the use of strict mapping parameters.

Reasons for the correct identification by the other two consensus methods (2/3 and MC) include: i) correct abundance threshold definition and ii) regions of multiple alleles in which by chance the correct base barely passes the threshold. On the other hand, incorrect identifications were mainly due to: i) regions of low coverage, ii) incorrect threshold definition, iii) regions of multiple alleles, and iv) multiallelic regions in which many reads from the mtpt mapped because of the very relaxed parameter of disabled seed.

We also observed that the strictest mapping is the best one to use for all methods, while the most relaxed mapping has the highest impact on the methods, particularly for phasing. The method least influenced by the mapping stringency is the 2/3, the MC method is only moderately influenced, and odintifier is the most variable. Out of the 334 positions, all three methods correctly identified 68 positions (mainly due to homozygous SNVs), and in only 2 positions all methods determined an incorrect base. Various cases of combinations of the tested methods being correct in various degrees in comparison to the other methods could also be identified, being due to a combination of the previously discussed reasons. Although the coverage-based methods are able to correctly call more positions than odintifier, the phasing-based method is able to resolve more positions coming from regions source of multiple odins that the coverage-based methods incorrectly call regardless of the mapping stringency (Additional file 1, Additional file 3: Table S2, Additional file 3: Figure S6).

**Fig. 5:**
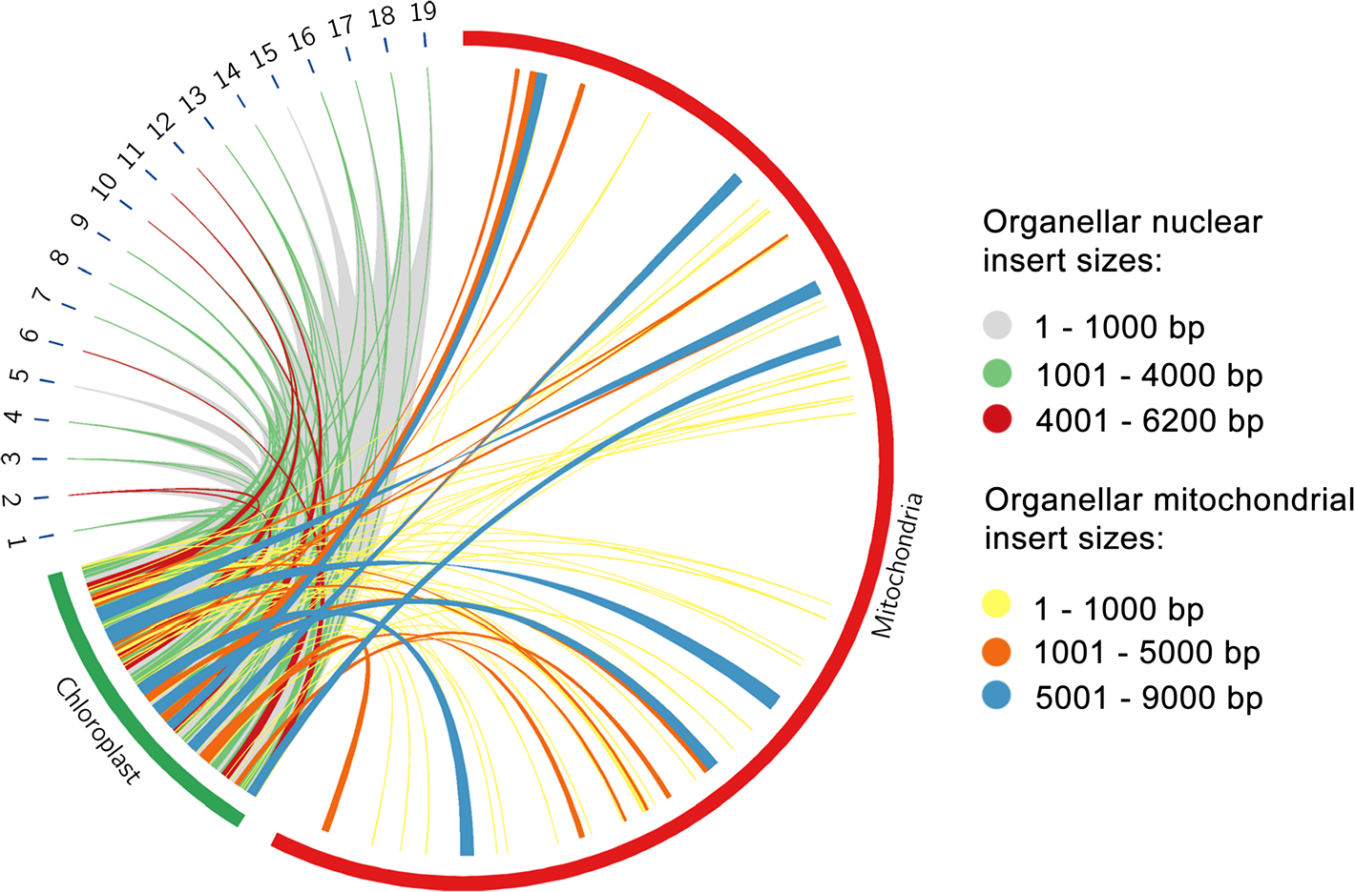
Characterization of grapevine odins. Distribution of the plastid inserted sequences into the nuclear and mitochondrial genome of grapevine. Only the length of the plastid and the mitochondria genome are proportional to their actual size

The large number of identified regions exhibiting various alternative alleles enabled us to profile the distribution of the plastid sequences inserted into the nuclear and mitochondrial genomes. We found that practically all the plastid genome has been inserted ubiquitously into every chromosome of the nuclear genome, and is also present on a large portion of the mitochondrial genome. The length of the inserted sequences ranges from a few hundred to 9,000 bps (Fig. 5, Additional file 3: Figure S7, Additional file 2).

In summary, this dataset represents the most complicated case that can be handled with a phasing-based assembly method. In spite of this, odintifier is able to accurately call many positions that are incorrectly called by the other coverage-based methods with any mapping strategy, thus fulfilling its goal of odin-aware assembly of the organellar genome sequence. However, given the nature of this dataset, as in the African lion dataset, the second goal of odintifier is not possible to be completely fulfilled.

## Discussion

We showcase the use of phasing for accurate reference-based assembly of organellar genomes and simultaneous identification of odin sequences. To this end, we used four different datasets of different complexities regarding the extent of organellar sequence insertions into other organellar genomes, and the integrity of the DNA extracted with different protocols (organelle-enriched and DNA shotgun sequencing from ancient and modern samples).

### Assessment of a de novo assembly sequence

The analysis of the bugle dataset showed that *de novo* assembly of an organellar genome that does not contain a significant percentage of odins is a viable approach. However, we highlight the risk of obtaining inaccurate sequences from reference-based assemblies based on coverage alone, even if an accurate sequence is used as reference, as shown by the sequence similarity dendrogram of the sequences assembled with the three tested methods on the bugle dataset (Fig. 2b). Even though the identified mtpt region in bugle is only 1,449 bps long, it contains a number of miscalled nucleotides on the reconstructed gene sequence from the coverage-based methods. Non-synonymous SNVs may lead to erroneous assumptions on the characteristics of the corresponding protein, and both non-synonymous and synonymous SNVs may influence results drawn from population studies.

### Iterative phasing to resolve multiallelic blocks

Despite the complications of analyzing datasets from species such as lions, that are known *a priori* to contain high levels of numts, our method was able to reconstruct accurate sequences, as suggested by the phylogenetic trees that place the mitochondrial sequences reconstructed with our phasing-based method together with the sequences used as reference. In the African lion dataset, the use of iterations helps to extend the phased blocks by allowing the mapping of reads that could not map before due to having many alternative nucleotides. However, iterations may still not be enough for very complicated cases in which a region in the assembly contains a high density of variants and does not have neighboring blocks that can be easily phased, as observed in the iterations 9–15 in the African lion dataset (Fig. 3a). To resolve this we performed iterations in which the reads are separated by identified allele in each phased region using the samtools-based approach instead of the GenomeAnalysisTK-based approach for performing the phasing step. This alternative strategy, as implemented in odintifier, filters out of the reads coming from the various alleles originated by the odin that provide confounding information for the phasing algorithm. Ultimately, this greatly enhances the resolution of multiallelic regions.

### Coverage normalization

Importantly, the analysis on the coverage from the different iterations performed on the African lion dataset showed that, as phasing rounds are performed, the coverage starts to be normalized across the reference sequence. In the first mapping there is a clear bias in the center of the reference, which exhibits coverage at approximately double the level observed at the extremes. As the iterations of the method increase, the coverage on the erstwhile doubled region decreases (Fig. 3b). This confirms that the reads derived from odins no longer map, thus are not artificially inflating the coverage. The reason for this might be that they do not pass the mapping thresholds of the number of mismatches to the reconstructed reference sequence, which is more accurate in each iteration. In this way, the fewer mapping reads correspond to the mitochondrial sequence that is present in abundance similar to the regions of the mitochondria that do not present many numt alleles.

Despite this, the control region represents a special case given that it is the most rapidly evolving region of the mitochondrial genome [63] and that it contains repetitive regions that can be heteroplasmic [64]. Since our method excludes indels from the VCF files because the current phasing algorithms are not able to properly deal with them, and given that it also includes a step of removing duplicate mapping reads, the control region presents uneven coverage in all the iterations. This suggests that the reconstructed control region sequence might not be as reliable as the rest of the sequence. If so, this could bias the phylogenetic reconstruction, thus we excluded it from the alignment.

With regards to the reconstruction of the *atp8* gene and pseudogene sequences from the lion aDNA dataset, it should be noted that we could not obtain the gene sequence when mapping against the pseudogene sequence DQ318555.1 because only few reads corresponding to the actual pseudogene mapped and thus no heterozygous positions were produced to be phased. This demonstrates that when aligning to a pseudogene sequence, some situations may arise in which only the pseudogene-derived reads map, thus conferring an artificial drop of coverage. The opposite case, reads derived from both the pseudogene and the gene mapping to a gene reference sequence, causes an artificial increase in the coverage as observed in the first iterations of the African lion dataset.

### Impact of the mapping stringency

Previous observations of bias on the mapping reads for detection of heterozygous SNVs toward higher mapping rates of the allele in the reference sequence [65] points to the use of different mapping strategies as an important aspect to consider. This also highlights the importance of implementing methods, such as odintifier, that are not coverage-based. The allowed number of mismatches between the read and the reference sequence is of particular importance for odintifier, given that its primary source of information are the called SNVs that can vary according to the mapping stringency. The importance of the mapping stringency is more evident on the grapevine and the African lion datasets, which have a large number of odins*.* It is interesting to note that as iterations proceeded in the African lion dataset, more relaxed mapping parameters on the last iterations helped resolve difficult multiallelic blocks by allowing more haplotype informative reads to map and produce more SNVs that could be used. However, the grapevine plastid poses a greater challenge due to the numerous multiallelic regions arising from the organellar sequences not only inserted into the mitochondria, but also into all nuclear chromosomes. In such cases, it is recommended to use very strict sequence similarity parameters for mapping the reads to the reference, thus preventing the mapping of the various odin-derived alleles that confound the phasing algorithms, which cannot yet properly handle polyploidy. Although the coverage in multiallelic regions could be inflated due to the mapping of odin-derived reads, the assumption that reads coming from the source organelle of interest will be in higher abundance than those coming from the other host organelle is correct in most cases and thus the correct nucleotide is identified with strict coverage-based thresholds, such as the 2/3 ratio. However, there might also be some cases in which this assumption is violated and the use of the linked SNV information is necessary to correctly identify the base.

aDNA datasets usually require relaxed mapping parameters in order to achieve a good coverage on the used reference sequence, which frequently comes from a distant relative to the ancient species under study. Thus, although we demonstrate how phasing can be successfully applied to both modern and aDNA datasets, and in doing so produce sequences with high accuracy, aDNA datasets from species known to have ubiquitous odins should be analyzed with much more attention. In such datasets, we suggest that the most accurate sequence could be obtained by combining the sequences obtained by phasing with the strictest mapping parameters and that from the 2/3 coverage-based methods. We suggest this based on the following observations. Firstly, phasing identified the largest amount of SNVs that were incorrect on all the other coverage-based methods under any mapping stringency (26 out of 31 of such positions) (Additional file 3: Figure S6, Additional file 3: Table S2). Secondly, one of the main reasons underlying why phasing called some nucleotides incorrectly were single SNVs, which could be called by a coverage-based method. Thirdly, the 2/3 method is the least influenced by mapping strategy, producing the largest number of correctly determined bases.

### Detection of numts on ancient samples

Even though the tested coverage-based methods were able to correctly assemble the *atp8* gene sequence from the lion aDNA dataset, they are unable to reconstruct the analogous numt sequence. Numts can be used to reconstruct ancestral sequences [42, 66], thus an integrative study of this type of analysis together with aDNA would provide an even deeper insight into the past. However, due to the degraded nature of aDNA, the study of numts directly from ancient samples has not been extensively performed. Although there are few reports of identification of numts on ancient samples [38], the reconstruction of ancient odins has not been considered of importance and studies mostly focus on the reconstruction of the mitochondrial genomes through the use of thresholds based on coverage. Here we showcase the identification and reconstruction of an *atp8* numt sequence in an ancient lion sample to highlight the importance of the use of methods not solely based on coverage (see Suppl Information for an extended discussion on odin sequence reconstruction). Furthermore, we hope that our method helps in opening the way to the development of studies using information from odins and aDNA in an integrative fashion.

### Ubiquitous organellar insertions

Similar to the African lion dataset, the grapevine dataset poses a great challenge to the sequence assembly using any of the conventional methods. Although the phasing-based method can correctly decide on many of the differing positions compared to the tested coverage-based methods, this dataset poses the extra challenge of organellar sequences being ubiquitously inserted many times, not only into the mitochondria, but also into the nuclear genome, thus the reads mapping to the source sequence will contain a lot of variation (Fig. 5, Additional file 3: Figure S4, Additional file 3: Figure S5). Multiple insertion events (and possible duplications of the inserted sequences) increase the ploidy-level and the diploid assumption on the phasing algorithm is violated. Considering these aspects, only the organellar sequence can be reliably recovered from multiallelic regions, such as those observed in the grapevine and in the Africa lion. In such cases it is not possible to confidently reconstruct the sequences of all the odin alleles, even with the use of various iterations of our method. This is due to the limitation of phasing algorithms to work optimally for only two alleles. The use of phasing algorithms capable of dealing with higher ploidy would aid in the recovery of more odin alleles, although the post processing of such an output would need to be accordingly modified. Currently, few phasing programs provide such capability [67], and future improvement of the phasing algorithms to enable them to accurately phase polyploidy datasets will aid resolution to this problem [68].

## Conclusions

To our knowledge, odintifier represents the first integration of phasing algorithms into a reference-based organellar genome sequence assembly method, that furthermore allows for the simultaneous identification and reconstruction of odin sequences. We applied our method on four datasets encompassing plants and felines to show that it is able to i) reconstruct the odin sequence, ii) reconstruct more accurate organellar genomes, iii) provide an objective reference-based assembly pipeline that does not require arbitrary coverage thresholds for calling the consensus sequence, and iv) be applicable to HTS datasets from modern as well as from aDNA. These datasets represent different levels of complexity regarding the quality of the DNA (modern and ancient), the extent of the occurrence of odins, and the method of the DNA sequencing (shotgun and organellar-enriched). Thus, we prove that our method can be applied on many kinds of datasets, without requiring any specific guideline for their generation. Although in general, as for many other types of analyses, longer read lengths are preferred, given that they would span a longer region that can contain SNVs, thus providing more haplotype informative reads for the phasing algorithm.

We demonstrated that odintifier can be used in mainly three different ways: 1) As an assembler for datasets with few odins, 2) as an assembler for datasets with multiple odins with the use of various iterations, 3) as a complementary aid together with de novo or coverage-based sequence assembly in order to provide an improved sequence and to identify regions source of odins. Finally, given the principle in which the method works, it could be potentially applied not only for reconstruction of odins, but also for disentangling sets of paralogous genes in nuclear genome assembly. It is hoped that with future improvement of phasing algorithms, their use in other non-conventional aspects such as that presented here will be more widely spread.

## Methods

### Data sources

We used four different datasets to validate odintifier and to prove the benefits that can be obtained from its use on real cases. First, in order to determine the quality of a *de novo* assembled plastid sequence and to simultaneously reconstruct the odin sequence we used the assembled chloroplast and dataset from the plant *Ajuga reptans* (bugle), published by Zhu et al. (2014) [35]. Briefly, organelle-enriched DNA was prepared by differential centrifugation and then 2 Gb of 100 bp paired-end reads from an 800 bp library were sequenced using the Illimina HiSeq2000 platform. Then, RNA isolation and RT-PCR were performed. The plastid sequence that we used as reference has the GenBank id NC_023102.1 and the mitochondrion sequence has the id NC_023103.1.

Secondly, in order to test odintifier on an assembled mitochondrial sequence from a feline species, a taxonomic group that is known to present a large number of numts [57], with the goal of assembling a mitochondrial sequence clean of odin-derived sequences, we used the dataset produced by Cho et al. (2014) [69] from *Panthera leo ssp.* using HiSeq2000 with read and insert lengths of ~90 bp and ~400 bp, respectively. For the analysis of this dataset with our phasing-based method, we used as reference sequences the assembled mitochondrial sequence generated by Ma and Wang (2014) [59] (KF776494) and that from *Panthera leo persica* (NC_018053.1 [60]).

Thirdly, in order to validate the use of odintifier on an aDNA dataset and obtain both the source and the inserted sequence, we used an unpublished dataset derived from an ancient sample of *Panthera leo leo* (Barnett et al. unpublished)*.* This data was generated from a fragment of male Barbary lion skull sampled from the Muséum National d’Histoire Naturelle in Paris (Accession Number 1931–582). We also used the reported gene sequence of *atp8* from *Panthera leo persica* (NC_018053.1 [60]) and from *Panthera leo spelaea* (DQ318556.1 [61]) as well as the corresponding numt sequence from *Panthera leo spelaea* (DQ318555.1 [61]) for the mapping step of this dataset.

Lastly, in order to test our method on the most complex possible dataset and evaluate the impact of the mapping stringency parameters compared to the other coverage-based methods, we applied odintifier on an aDNA dataset from a common grapevine (*Vitis vinifera*). This is a species with a very large proportion of ptDNA inserted into the mitochondrial [62] and the nuclear genomes [70], thus containing many regions with very large amounts of alleles. Such regions significantly challenge the accurate sequence reconstruction by the method. The DNA library of this dataset was enriched for chloroplast DNA with an approach that led to the enrichment of genuine chloroplast DNA, as well as mtpts and nupts (See Suppl Information for further details). For the mapping step we used two different reference sequences: i) the plastid genome (NC_007957.1 [71]) and ii) the nuclear (*Vitis vinifera* assembly 12X, GenBank assembly accession GCA_000003745.2) and mitochondrial (NC_012119.1 [62]) genomes together.

### Data pre-processing

Firstly, adapter sequences and low quality bases were removed from the reads. Subsequently, the cleaned reads were mapped against their corresponding reference sequence with bwa [72] (see Suppl Information for details). We refer to this reference sequence used for the mapping as primary reference. Next, the bam file of each dataset was sorted with samtools v 0.1.18 [73], and duplicates were detected with Picard v1.92 [74] for subsequent removal. The resulting bam files were realigned with GenomeAnalysisTK v2.8 [75, 76]. SNV calling was then performed with GenomeAnalysisTK UnifiedGenotyper with -stand_call_conf 30.0, −stand_emit_conf 10.0, −glm SNP, −dcov 300. Finally, diploid phasing was performed with GenomeAnalysisTK ReadBackedPhasing with --phaseQualityThresh 10.0. Resulting alignments were visualized with IGV v2.2.11 [77].

Due to the presence of various numt-generated multiallelic regions in the African lion dataset, we also used a second strategy for SNV calling and phasing, consisting of using samtools instead of GenomeAnalysisTK. Specifically, we used samtools calmd to generate the MD tag followed by samtools phase with -F so that chimeras are not fixed, given that they could represent an odin. We performed a total of 31 iterations, 26 with the regular GenomeAnalysisTK-based procedure, and 5 using the alternative samtools-based approach (one after regular iteration 15, 20, 23, 24, and 25). Out of the five iterations of mapping followed by samtools for phasing, we used bwa with -n 0.001 for the first three, and a more relaxed -n 0.0001 for the last two.

### Method development

A python script was made to process the phased SNVs from the mapping against the primary reference, producing two sequences per phased region. Another script was developed in R v2.15.3 [78] using the package Biostrings v2.26.3 [79] to align the masked phased block sequences to another reference sequence, which we will refer to as the secondary reference sequence (see Suppl Info for further details on the distinction of primary and secondary reference sequence).

Afterwards, based on the alignment score to the secondary reference, the origin of the sequences from each region is determined as either organellar or odin. The output of this script is the final phased consensus sequences of the organellar genome and the odin. Finally, using bedtools v2.17.0 [80] the phased regions with coverage less than 1 are masked.

Datasets containing a large number of multiallelic blocks can undergo various iterations of mapping, SNV calling, phasing, and post processing until the recovered sequences do not significantly change from one iteration to the next. Afterwards, further iterations following a different procedure in which we use samtools for SNV calling and phasing as previously described on the African lion dataset can be performed to boost the accuracy of the sequence reconstruction. To this end, odintifier sorts the reads from the two bams generated by samtools into those coming from the organellar genome and those coming from the odin. The scripts of the method are available at https://github.com/SamaZYX/odintifier.

### Majority count and 2/3 ratio assembly

The performance of odintifier was compared against two other widely used reference-based assembly methods based on coverage. To do this, we used GenomeAnalysisTK with the VCF generated in the SNV calling step filtered by two abundance criteria: 1) “MC”, in which the most common nucleotide is kept for the reference, and 2) “2/3”, an approach in which the alternative nucleotide is kept when present at a minimum ratio of 2/3. Finally, positions with coverage less than 1 on the assembled sequence were masked.

### Analysis of reconstructed sequences

Firstly, in order to characterize the mtpt from bugle, the reconstructed odin sequence was translated with transeq EMBOSS v6.4.0.0 [81] using the translation table 11 and non-synonymous SNVs were identified. Afterwards, sequence similarity dendrograms of the assembled sequences from the two lion datasets and bugle were obtained. To this end, the sequences were aligned with MUSCLE v3.8.31 [82] and the control region was removed from the African lion alignment*.* Then, a phylogenetic tree was constructed with MEGA v5.2.2 [83] using the neighbor-joining pair-wise distance method with 100 bootstrap replicates for all the datasets.

Furthermore, in order to characterize the mapping stringency impact on the tested coverage and phasing-based methods, the grapevine dataset was mapped against the plastid reference genome (NC_007957.1 [71]) in four different ways: 1) a relaxed one disabling the seed, 2) default parameters, 3) strict one of -n 0.1, and 4) a stricter of -n 2. The assembled sequences from the four different mappings were aligned with MUSCLE [82]. Then, we manually characterized each of the differing positions between the 12 assembled sequences compared to the reported reference sequence of grapevine chloroplast. To aid the manual characterization of positions in regions of multiple alleles, we also mapped the reads against the concatenated fasta of the nuclear, mitochondrial and plastid genomes as primary reference with mapping stringency of -n 2.

Lastly, in order to characterize the distribution of the odins of plastid origin in grapevine, we used LASTZ v1.02.00 [84] with the default parameters to align its plastid genome (NC_007957.1 [71]) to the nuclear (*Vitis vinifera* assembly 12X, GenBank assembly accession GCA_000003745.2) and mitochondrial (NC_012119.1 [62]) genomes. The distribution of the translocations was visualized with circos v0.63 [85].

## Additional files

Additional file 1:
**This file contains the manual characterization of the 334 differing positions of the 12 sequences assembled with the grapevine dataset.** (XLS 87 kb) Additional file 2:
**This file contains the coordinates of the identified sequences from plastid origin inserted into the nuclear and mitochondrial genomes in grapevine.** (TXT 301 kb) Additional file 3:
**This file contains the supplementary figures, tables, and extended information from the methods and discussion sections.** (PDF 1023 kb)
