# Supplementary material for: Odintifier - A computational method for identifying insertions of organellar origin from modern and ancient high-throughput sequencing data based on haplotype phasing
Source: BMC Bioinformatics. 2015 Jul 28;16(1):232. doi: 10.1186/s12859-015-0682-1 (PMC4517485; doi:10.1186/s12859-015-0682-1)
Supplement: Additional file 3: — This file contains the supplementary figures, tables, and extended information from the methods and discussion sections. (PDF 1023 kb) [file 12859_2015_682_MOESM3_ESM.pdf]

Supplementary Information

Supplementary figures

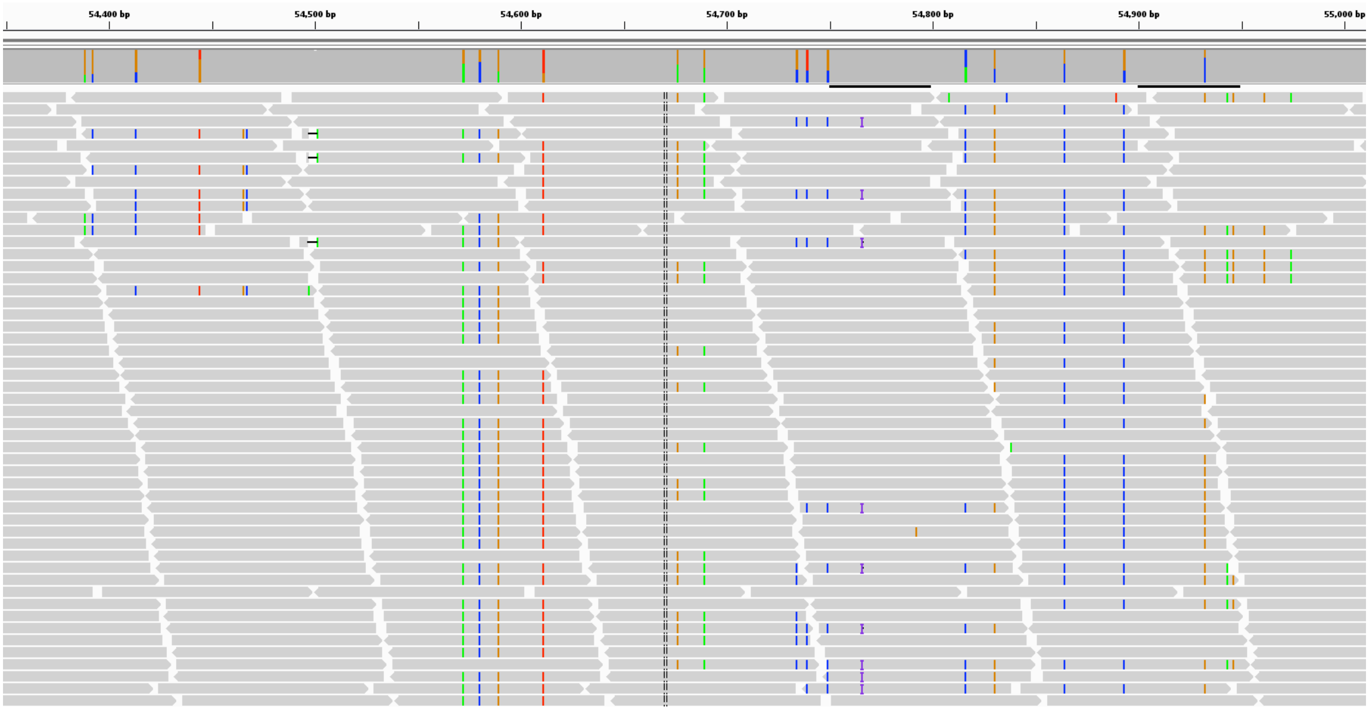

Suppl Fig 1 *Ajuga reptans* phased block. Only one region was identified as containing reads from the odin corresponding to a fraction of the gene *rbcL*. Reads corresponding to the pseudogene contain alternative nucleotides while those from the chloroplast do not contain any variability.

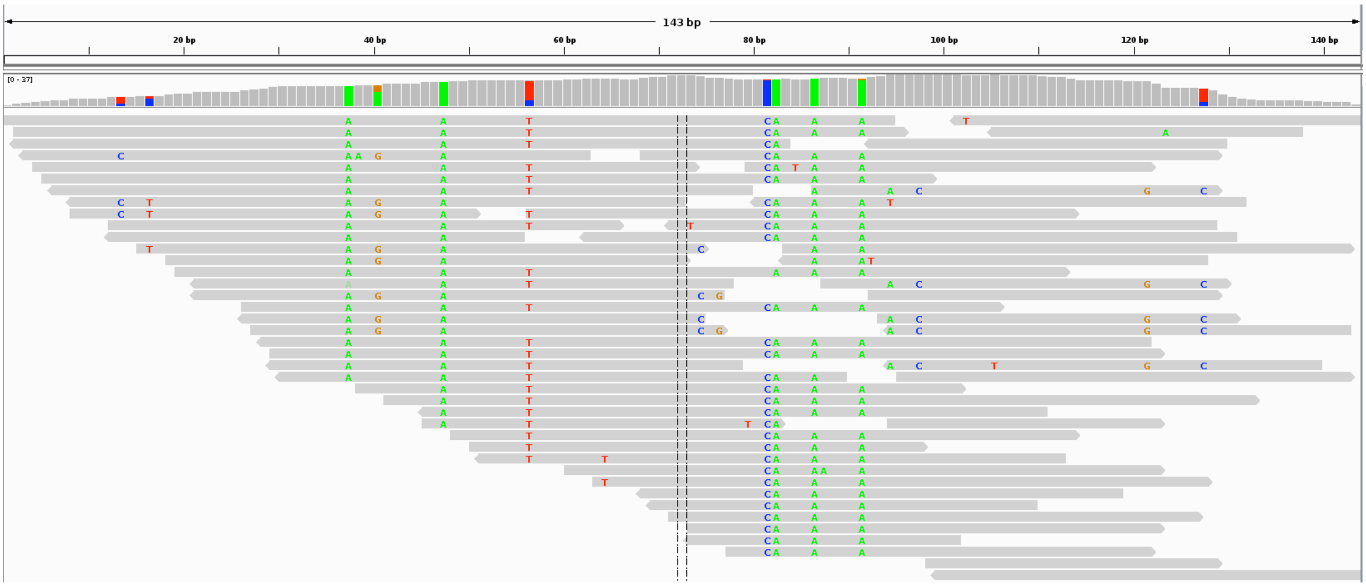

Suppl Fig 2 *atp8* from *P leo leo*. Reads from numt and mitochondrial gene origin mapping to the well characterized *atp8* organellar gene reference sequence DQ318556.1 from *Panthera leo spelaea.*

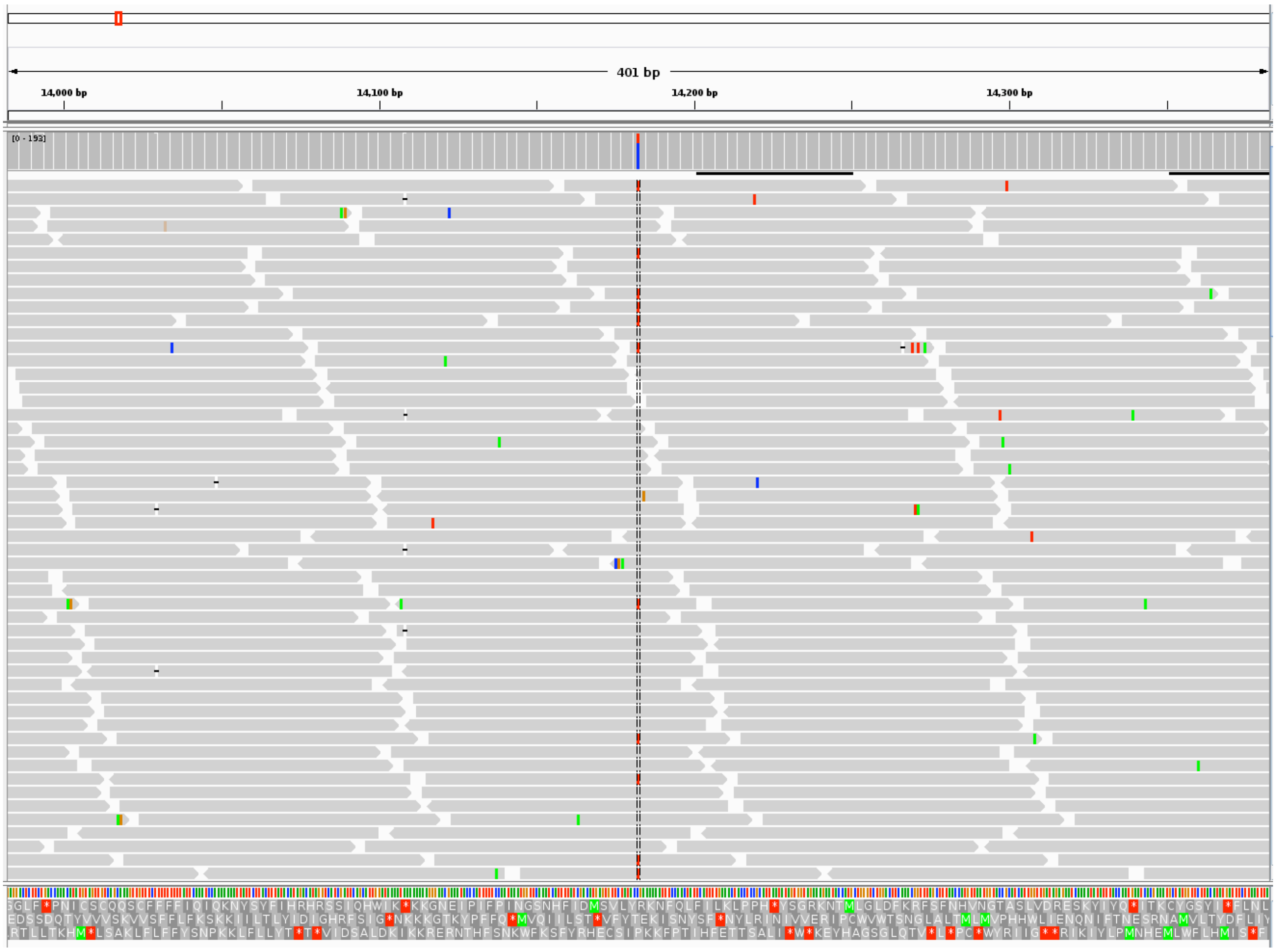

Suppl Fig 3 Case example from the grape dataset of a single SNV that has no surrounding haplotype informative reads. The phasing algorithm works on linked SNVs, thus when there is only one SNV with no surrounding SNVs the algorithm does not identify it. Coverage-based methods can be used to deal with these cases.

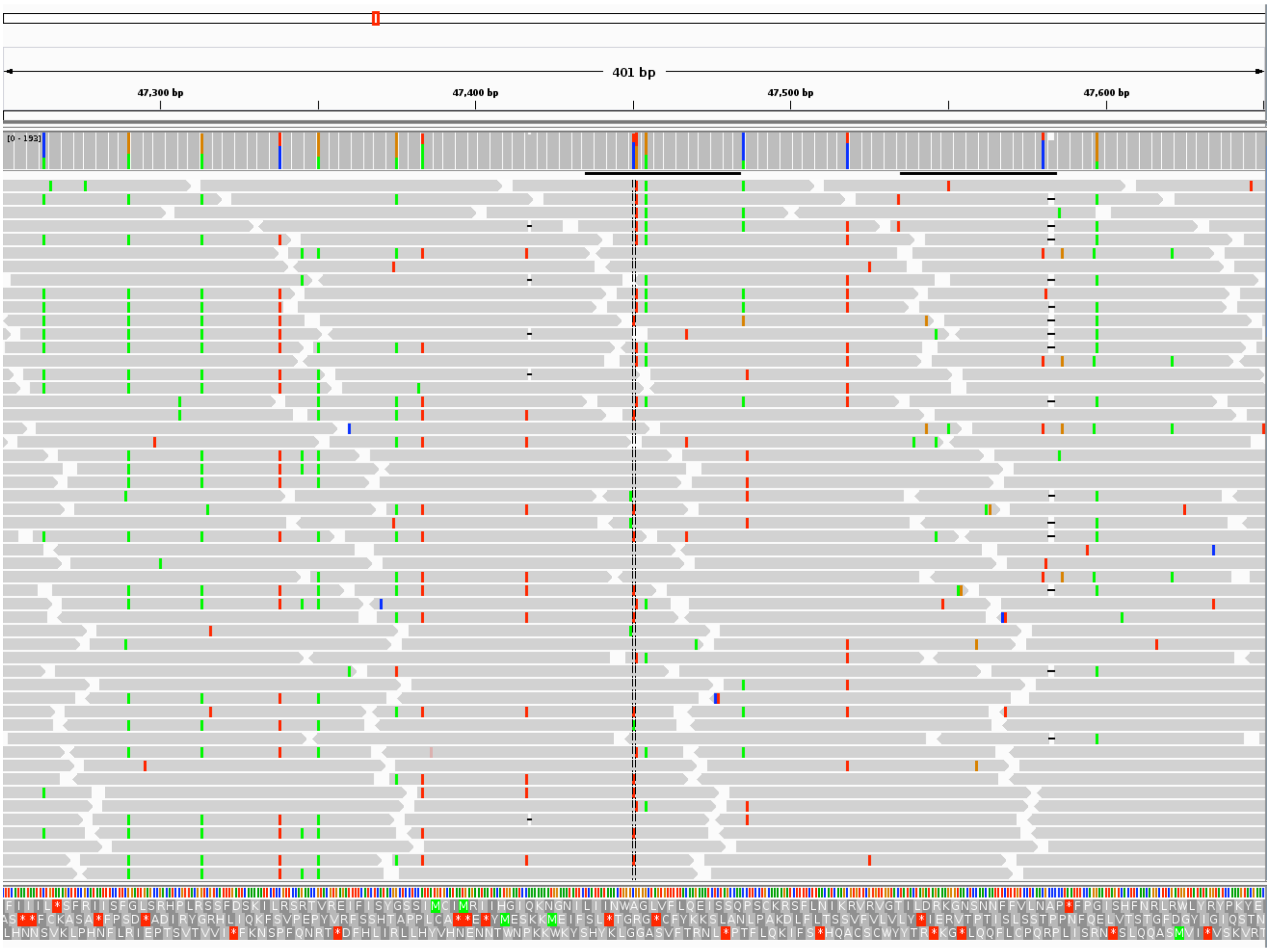

Suppl Fig 4 Case example of the grape dataset of a chimeric block arisen by a region with multiple alleles. The identified phased block that goes from 47092-47936 is a multi-allelic block in which a chimera is produced, with the switch point at position 47450 (red SNV, shown in the marked column in middle of this image). From the phasing results, only the one using the strictest mapping stringency could correctly call this position. The region at the right of position 47450 was being phased correctly calling the two present alleles (allele0 and allele1, corresponding to chloroplast and odin, respectively); however, at position 47450 a third allele arises (see the green SNVs immediately next to 47450) and disappears soon after to leave the right side of the block with again only two alleles. This causes the allele0 from the left side to continue being phased with the allele0 from the right side that now corresponds to the odin sequence, instead of to the chloroplast as on the left side of the block. Likewise, allele1, which on the left side corresponds to the odin, on the right side corresponds to the chloroplast. Thus, the final phased sequence of that block contains half chloroplast and half odin sequence.

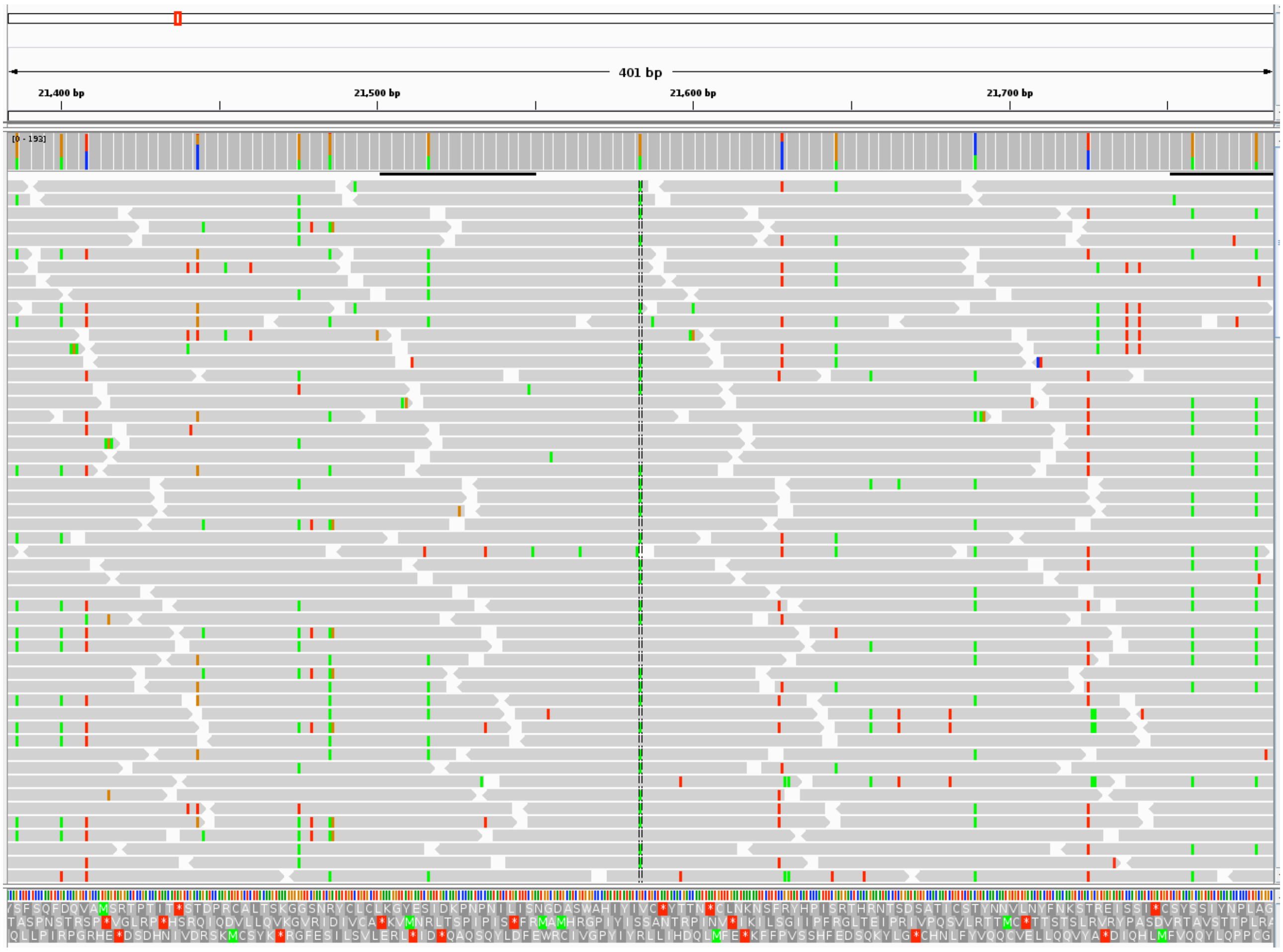

Suppl Fig 5 Case example from the grape dataset of a multiallelic block. The base highlighted in the middle of the image corresponds to position 21583. From the phasing results, only the one using the strictest mapping parameters could correctly identify it. The main confounding reason for the phasing are positions 21596, 21627, 21628, and 21629, that contain SNVs corresponding to more than 2 alleles.

	Method 1				Method 2				Method 3			
Category	Mapping1	Mapping2	Mapping3	Mapping4	Mapping1	Mapping2	Mapping3	Mapping4	Mapping1	Mapping2	Mapping3	Mapping4
1	✓	✓	✓	✓	✗	✗	✗	✗	✗	✗	✗	✗
2	✓	✓	✓	✗	✗	✗	✗	✗	✗	✗	✗	✗
3	✓	✓	✗	✗	✗	✗	✗	✗	✗	✗	✗	✗

Suppl Fig 6 Categories of the grouped nucleotide differences from the reconstructed references. For each method, the observed nucleotide differences between the reconstructed sequences with the four mapping stringency thresholds are grouped into three categories: the base assigned to

the position is correct in the method on all (category 1), at least three (category 2), and less than three (category 3) of the mappings while incorrect in all the other mappings of the other methods.

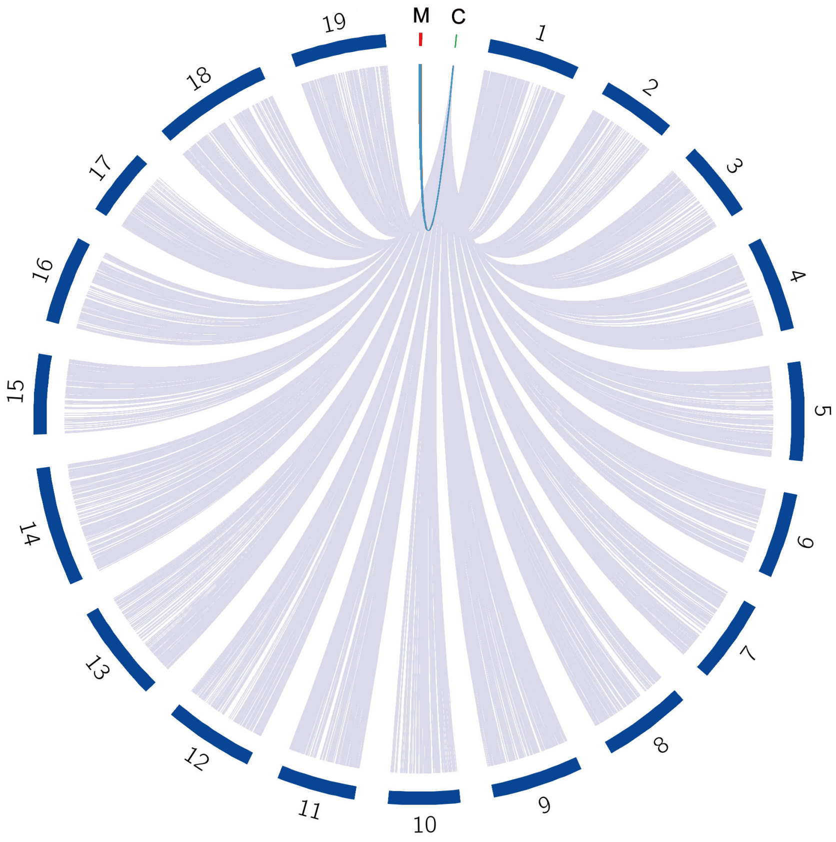

Suppl Fig 7. Sequence translocations from the chloroplast (C) into the mitochondria (M) and nuclear chromosomes in *Vitis vinifera*. The length of the chromosomes, mitochondria and plastid are proportional to their actual length.

Supplementary Tables

Suppl Table 1 Identified odin-source regions in the African lion mitochondria (KF776494.1). The coordinates derive from merging the overlapping identified phased blocks from all the performed iterations.

Start	End
430	499

4450	4656
4795	4827
5040	6145
6261	7365
7420	8217
8319	8553
8608	10654
10780	10900
11000	11608
16353	16434

Suppl Table 2 Correctly determined positions using the three tested methods with four different mapping strategies

Method	Category*			Mapping stringency				Main reason	
	1	2	3	Relaxed	Default	Strict	Strictest	Correct call	Incorrect call
Phasing	9	10	7	191	236	236	294	Linked SNVs information	Multi-allelic or chimeric block, single SNVs
MC	0	0	0	255	277	277	297	Homozygous position, correct coverage threshold definition	No use of linked SNV information, incorrect threshold definition, multi-allelic block

2/3	2	2	1	295	302	303	309	Stricter 2/3 coverage-based better than only MC, homozygous position	No use of linked SNV information, incorrect coverage threshold definition, multi-allelic block

* Categories 1-3 correspond to: 1) the base assigned to the position is correct in the method on all four, 2) three or four, and 3) one or two of the mapping strategies, respectively, while incorrect in all the other mappings of the other two methods. See Suppl Fig 6 for a visual explanation

Supplementary Information

Supplementary methods - Data pre-processing

Sequence adaptors were trimmed from the reads from the *A. reptans* dataset using Trimmomatic v0.32 [1] keeping the reads with a minimum length of 40 nts. Then PRINSEQ-lite v0.20.3 [2] was used to remove reads with a minimum of 10 Ns, low quality reads were trimmed if having a score less than 30 on the left and 20 on the right side, and reads were discarded if having a mean read quality less than 20 and a minimum length of 25. Reads from the grapevine and the *Panthera leo leo* datasets were cleaned with AdapterRemoval v1.5.4 with mm 3, trimming the Ns in the extremes, and trimming bases on the extremes with quality less than 33, and minimum length of 25.

The cleaned reads were mapped against their corresponding organellar reference sequence with bwa v0.6.2-r126 [3] samse. In *A. reptans* the seed was disabled in order to be relaxed in the mapping and to recover the sequences from the pseudogene. Given the aDNA nature of the *P. leo leo* dataset, it was mapped disabling the seed with -n 0.001. In order to test the effect of the mapping stringency on the efficiency of our method, the *V. vinifera* dataset was mapped in 4 different ways: 1) a relaxed one disabling the seed, 2) default parameters, 3) strict one of -n 0.1, and 4) a stricter of -n 2.

Grapevine dataset

This dataset comes from a common grapevine sample collected in Areni, Armenia. A leaf disc was extracted following the method described by Japelaghi et al. (2011) [4]. Then, the DNA was converted to an Illumina sequencing library (New England BioLabs, Ipswich, MA, product number

E6070L), and the library was enriched for chloroplast DNA using a custom-designed MYbaits in-solution capture kit (MYcroarray, Ann Arbor, MI) (personal communication, Nathan Wales et al.). The enriched library was sequenced on an Illumina HiSeq in 100bp single read mode.

Primary and secondary reference sequences

The secondary reference sequence may or may not be the same as the primary sequence; it would depend on the interest of the user. For example, if the user is more interested in accurately assembling the odin sequence, for instance an mtpt, then the secondary reference can be the mitochondrial region that is the host of the odin, while the primary reference is the plastid genome. On the other hand, if the goal is the accurate reconstruction of the plastid genome, then both the primary and the secondary references should be the plastid sequence. Although odintifier will also simultaneously reconstruct the odin sequence when using the primary sequence as the secondary sequence, the use of a different secondary reference would ensure a more accurate odin reconstruction, specially in cases where the odin source sequence is known to be inserted in multiple places in the host genome. When performing various iterations with odintifier, the secondary reference sequence can be the same as the primary reference sequence in the first iteration, but in following iterations the primary reference sequence should be the consensus sequence resulting from the previous iteration.

Odin sequence reconstruction

While odintifier can correctly reconstruct the gene sequence, the pseudogene might not be completely reconstructed with as much accuracy as the gene when using only one iteration. This could happen when the mapping stringency prevents the mapping of more haplotype informative reads on the extremities of the odin block to extend the phased pseudogenic sequence. This can be due to the mitochondrial sequence having different rate of evolution from the plastid, so that the number of mismatches on the pseudogene is larger than the ones allowed by the mapping parameters, causing the odin reads not to map. Since only few odin reads can map, the frequencies of the alternative bases are very low that they are not considered by the SNV calling program. Given that phasing uses the called SNVs, it will not be able to phase a block in which there is no information. If the pseudogene needs to be reconstructed with better accuracy, more rounds of iterations should be performed. In these iterations, the primary and secondary reference sequences to use should contain the odin-phased sequence obtained from the previous round in that block instead of the gene sequence, thus more pseudogenic reads will map to that region and extend the phased block.

References

1. Bolger AM, Lohse M, Usadel B. Trimmomatic: a flexible trimmer for Illumina sequence data. Bioinformatics. 2014;30:2114–20

2. Schmieder R, Edwards R. Quality control and preprocessing of metagenomic datasets. Bioinformatics. 2011;27:863–4

3. Li H, Durbin R. Fast and accurate short read alignment with Burrows-Wheeler transform. Bioinformatics. 2009;25:1754–60

4. Japelaghi RH, Haddad R, Garoosi GA. Rapid and efficient isolation of high quality nucleic acids from plant tissues rich in polyphenols and polysaccharides. Mol. Biotechnol. 2011;49:129–37
